# TMPRSS4: A Novel Tumor Prognostic Indicator for the Stratification of Stage IA Tumors and a Liquid Biopsy Biomarker for NSCLC Patients

**DOI:** 10.3390/jcm8122134

**Published:** 2019-12-03

**Authors:** Maria Villalba, Francisco Exposito, Maria Jose Pajares, Cristina Sainz, Miriam Redrado, Ana Remirez, Ignacio Wistuba, Carmen Behrens, Eloisa Jantus-Lewintre, Carlos Camps, Luis M. Montuenga, Ruben Pio, Maria Dolores Lozano, Carlos de Andrea, Alfonso Calvo

**Affiliations:** 1 IDISNA and Program in Solid Tumors, Center for Applied Medical Research (CIMA), University of Navarra, 31008 Pamplona, Spain; mvillalbae@unav.es (M.V.); fexposito@alumni.unav.es (F.E.); mjose.pajares@unavarra.es (M.J.P.); mcsainz@unav.es (C.S.); miredrado@unav.es (M.R.); aremirez@unav.es (A.R.); lmontuenga@unav.es (L.M.M.); rpio@unav.es (R.P.); ceandrea@unav.es (C.d.A.); 2 Department of Pathology, Anatomy and Physiology, School of Medicine, University of Navarra, 31008 Pamplona, Spain; mdlozano@unav.es; 3 CIBERONC, ISC-III, 28029 Madrid, Spain; jantus_elo@gva.es (E.J.-L.); camps_car@gva.es (C.C.); 4 Department of Translational Molecular Pathology, The University of Texas MD Anderson Cancer Center, Houston, TX 77030, USA; iiwistuba@mdanderson.org (I.W.); cbehrens@mdanderson.org (C.B.); 5 Molecular Oncology Laboratory, FIHGUV & Department of Biotechnology, Universitat Politècnica de València, 46022 Valencia, Spain; 6 Department of Medicine, Universitat de Valencia, 46022 Valencia, Spain; 7 Department of Biochemistry and Genetics, School of Sciences, University of Navarra, 31008 Pamplona, Spain; 8 Department of Pathology, University of Navarra Clinic, 31008 Pamplona, Spain

**Keywords:** NSCLC, TMPRSS4, liquid biopsy, prognosis, DNA methylation

## Abstract

Relapse rates in surgically resected non-small-cell lung cancer (NSCLC) patients are between 30% and 45% within five years of diagnosis, which shows the clinical need to identify those patients at high risk of recurrence. The eighth TNM staging system recently refined the classification of NSCLC patients and their associated prognosis, but molecular biomarkers could improve the heterogeneous outcomes found within each stage. Here, using two independent cohorts (MDA and CIMA-CUN) and the eighth TNM classification, we show that TMPRSS4 protein expression is an independent prognostic factor in NSCLC, particularly for patients at stage I: relapse-free survival (RFS) HR, 2.42 (95% CI, 1.47–3.99), *p* < 0.001; overall survival (OS) HR, 1.99 (95% CI, 1.25–3.16), *p* = 0.004). In stage IA, high levels of this protein remained associated with worse prognosis (*p* = 0.002 for RFS and *p* = 0.001 for OS). As *TMPRSS4* expression is epigenetically regulated, methylation status could be used in circulating tumor DNA from liquid biopsies to monitor patients. We developed a digital droplet PCR (ddPCR) method to quantify absolute copy numbers of methylated and unmethylated CpGs within the *TMPRSS4* and *SHOX2* (as control) promoters in plasma and bronchoalveolar lavage (BAL) samples. In case-control studies, we demonstrated that *TMPRSS4* hypomethylation can be used as a diagnostic tool in early stages, with an AUROC of 0.72 (*p* = 0.008; 91% specificity and 52% sensitivity) for BAL and 0.73 (*p* = 0.015; 65% specificity and 90% sensitivity) for plasma, in early stages. In conclusion, TMPRSS4 protein expression can be used to stratify patients at high risk of relapse/death in very early stages NSCLC patients. Moreover, analysis of *TMPRSS4* methylation status by ddPCR in blood and BAL is feasible and could serve as a non-invasive biomarker to monitor surgically resected patients.

## 1. Introduction

Management of lung cancer, the leading type of cancer worldwide, is still challenging, and mortality rates did not substantially decrease in the latest years [1]. Lung cancer is often diagnosed at advanced stages, when curative options are limited. When diagnosed in early stages, patients with lung cancer can be successfully treated with surgery. Nonetheless, recurrence rates after complete surgical resection are between 30% and 45% within five years of diagnosis [2]. Therefore, identification of factors that could predict patients at risk of recurrence after surgery is necessary for a better management of the disease. Currently, the tumor-node-metastasis (TNM) staging system is the only routine method to estimate prognosis in non-small-cell lung cancer (NSCLC) patients. However, in spite of the new classification (eighth edition [3]), survival prediction is not totally accurate, as different clinical outcomes are observed in patients within the same TNM stage. It was suggested that molecular biomarkers could help identifying patients with poor prognosis based on biological malignant features [4], although incorporation of such biomarkers into the clinical practice remains to be implemented.

Transmembrane protease serine 4 (TMPRSS4) is a member of the type II transmembrane serine protease (TTSP) family of genes, located in the long arm of chromosome 11 (11q23.3). TMPRSS4 is highly upregulated in solid tumors (including NSCLC), where it plays a role in facilitating the growth and metastatic spread of cancer cells [5,6]. Upregulation and association with poor prognosis were described for several cancer types, including NSCLC [7], pancreas [8], breast [9,10], and esophageal cancer [11]. The high expression of *TMPRSS4* in tumors is a consequence of aberrant hypomethylation, which is also associated with poor prognosis in NSCLC patients [7]. TMPRSS4 provides cancer stem cell (CSC) properties to lung tumor cells and makes them resistant to chemotherapy [5]. We previously showed in animal models that abrogation of TMPRSS4 using short hairpin RNA (shRNA) strategies impedes tumor homing and growth [5], suggesting that targeting this protein in NSCLC may result in a strong therapeutic effect. Two TMPRSS4-specific compounds were recently shown to inhibit protein activity and tumor growth in prostate cancer models [12]. Therefore, TMPRSS4 is an emerging candidate biomarker and therapeutic target in NSCLC patients.

The fact that *TMPRSS4* expression is epigenetically regulated by DNA methylation suggests that methylation status could be used as a biomarker in liquid biopsy through analysis of circulating tumor (ct)DNA. Liquid biopsy-based assays are used for diagnosis, prognostication, and monitoring of lung cancer [13]. One important advantage of methylation-based biomarkers is that DNA methylation is a highly stable covalent modification that occurs early during tumor progression and can be detected in fluids by PCR methods. Although the fraction of ctDNA obtained from fluids is often low (<1.0%), highly efficient amplification methods can accurately quantify methylation changes. Digital droplet PCR (ddPCR) is an ultrasensitive technology in which PCR reactions are partitioned in thousands of individual reactions, and it allows absolute quantification of the number of abnormal target DNA. This technique is used to quantify extremely low numbers of DNA copies in liquid biopsies. In lung cancer, ddPCR is used mainly to detect actionable mutations (such as *EGFR*) in cell-free DNA from plasma samples [14]. However, studies using ddPCR to quantify epigenetic alterations in fluids from NSCLC patients are lacking.

In this study, we addressed whether protein expression of the type II transmembrane serine protease TMPRSS4 could be used as a prognostic indicator in NSCLC tumors, following the eighth TNM classification. In addition, we assessed whether *TMPRSS4* promoter methylation status could serve as a biomarker in plasma and bronchoalveolar lavage (BAL) samples to differentiate between NSCLC patients and healthy controls. We also evaluated methylation status of the short stature homeobox 2 (*SHOX2*), as aberrant hypermethylation of this gene was proven in different studies as a reliable diagnostic biomarker in plasma [15,16,17] and bronchial aspirates [18] and is currently in clinical development.

Using a large cohort of patients and a validation cohort, we show here that TMPRSS4 protein expression is an independent prognostic factor in NSCLC, mainly in stage IA. In addition, we developed a robust ddPCR method to quantify CpG methylation levels within the *TMPRSS4* and *SHOX2* promoters in blood and BAL that can differentiate between NSCLC patients and tumor-free individuals.

## 2. Materials and Methods

### 2.1. Cell Lines

Lung cancer cell lines H2170, H1703, COR-L88, and LXF-289 were cultured in RPMI 1640 Glutamax supplemented with 10% HyClone Fetalclone III (Thermo Fisher, Waltham, MA, USA) and 1% penicillin–streptomycin (Lonza, Bruguières, France), at 37 °C in a 5% CO_2_ humidified atmosphere. All cell lines were periodically tested with the MycoAlert Mycoplasma Detection Kit (Lonza), to use mycoplasma-free cells.

### 2.2. Cohort of Patients for Immunohistochemical Analysis of TMPRSS4

Samples from primary lung cancer were collected from surgical specimens obtained at the University of Texas MD Anderson Cancer Center (Houston, TX) (MDA cohort) and CIMA-Clinica Universidad de Navarra (Pamplona, Spain) (CIMA-CUN cohort). Inclusion criteria were as follows: patients with complete resection of the primary tumor and absence of chemo or radiotherapy treatment prior to surgery. Lung tumors were classified according to the World Health Organization 2004 classification, and the eighth TNM edition was used for tumor stratification [3]. The MDA cohort was composed of 489 lung cancer patients diagnosed from 2006 to 2009 at the MDA. The CIMA-CUN cohort contained 95 patients diagnosed from 2000 to 2013. Reported recommendations for tumor marker prognostic studies (REMARK) criteria were followed [19]. This study was conducted according to the Declaration of Helsinki, and was approved by the Institutional Review Boards and Ethical committees of the participating institutions. Written informed consent was obtained from each patient. Detailed clinical and pathological information of the cohorts is summarized in Table 1.

### 2.3. Cohort of Patients for Methylation Analysis of TMPRSS4

To evaluate the methylation status of *TMPRSS4* and *SHOX2* by ddPCR in tissue specimens, a cohort of 59 patients from Hospital General Universitario de Valencia (HGUV) was used (Table S1, Supplementary Materials). This cohort included malignant and adjacent non-malignant tissue from patients mostly with squamous cell carcinomas (SCCs), stages I–III. Samples were obtained and snap-frozen at −80 °C until use.

Bronchoalveolar lavages (BAL) included 79 samples from patients with lung cancer and 26 tumor-free controls obtained from CIMA-CUN. Stage I–IV adenocarcinomas (ADCs), SCCs, and few cases from other histological types were included in this cohort (Table S2, Supplementary Materials). Upon collection, samples were centrifuged and stored in cryovials at −80 °C until use. In available cases, an aliquot of each sample was smeared on a slide to determine the presence of malignant cells.

Plasma samples were obtained from the University of Navarra Biobank, 89 of which corresponded to patients with NSCLC and 25 to healthy individuals. This cohort included ADC and SCC from stage I–IV NSCLC patients (Table S3, Supplementary Materials). Plasmas were processed within 2 h of blood extraction and frozen at −80 °C. Studies were approved by the Ethical and Scientific Committee of the University of Navarra with written informed consent from the patients (project number: 13/2016).

The characteristics of control individuals and patients for the study of BAL and plasma samples were similar between both groups in terms of age, sex, and smoking habits.

### 2.4. Evaluation of TMPRSS4 Expression by Immunohistochemistry

For immunohistochemistry, tissue microarrays (TMAs) containing three representative tissue cores per case were built. Slides were deparaffinized and rehydrated. Endogenous peroxidase was blocked with 3% hydrogen peroxide, and antigen retrieval was performed by heating the samples in a microwave oven using citrate buffer (10 mM, pH 6). After incubation with the primary antibody overnight at 4 °C with a previously validated anti-TMPRSS4 antibody from Ingenasa Inc., (1:500) [20], the Advance™ HRP system (Dako, Glostrup, Denmark) was used for detection of the signal. Finally, slides were counterstained with hematoxylin, dehydrated, and cover-slipped with DPX mounting medium (VWR, Barcelona, Spain). Slides were scanned with the Aperio CS2 scanner (Leica, Barcelona, Spain) at 20× magnification, and images were visualized with the Aperio Image Scope (v12.1.05029). Scores were established by semiquantitative analysis as previously described [21]. Briefly, staining was evaluated by two observers independently (M.J.P. and C.S.) unaware of the clinical features of patients. The extension was scored as percentage of positive cells (0–100%) and the intensity of staining (1+, weak; 2+, moderate; 3+, strong). An H-score was established for each patient using extension and intensity parameters. Both the median and quartiles were tested as cut-off values to define high/low TMPRSS4 expression levels.

### 2.5. DNA Isolation and Bisulfite Conversion

DNA from tissues and cells was isolated with the NucleoSpin TissueTM kit (Macherey-Nagel). We used for each sample five slides with 3-μm-thick paraffin-embedded tissue. For BAL and plasma, a 1-mL sample from each patient was processed, and DNA was isolated with the QiaAmp Circulating Nucleic Acid (Qiagen, Germantown, MD, USA) and the QiaAmp DNA Blood (Qiagen), following the protocols provided by the kits. The bisulfite conversion was carried out with the EZ-96 DNA Methylation-Lightning™ Kit (Zymo Research, Irvine, CA, USA). The ddPCR reactions were performed within 48 h post bisulfite conversion to reduce the possibility of DNA degradation.

### 2.6. Digital Droplet PCR to Detect the Methylation Status of TMPRSS4 and SHOX2

For the ddPCR, specific probes to identify either the methylated (labeled with FAM) or the unmethylated (labeled with HEX) CpGs were synthesized (Table S4, Supplementary Materials). Probes were 20–24 nt long, contained the CpG of interest in the middle of the sequence, and were devoid of SNPs. Primers flanking the probes were common for the methylated/unmethylated sequences and did not contain any CpG. For *TMPRSS4*, the CpG located at −70 bp upstream of the transcription start site (cg25116503 probe from the Infinium 450k methylation array) was evaluated. This CpG was selected based on our previous study showing strong hypomethylation in NCSLC specimens in comparison with non-malignant lungs [7]. In the case of SHOX2, the selected CpG was based on previous publications that used qMSP-PCR in plasma to differentiate healthy individuals from patients with NSCLC [18]. Primers and probes for ddPCR were designed according to Bio-Rad recommendations (http://www.bio-rad.com).

The QX200™ Droplet Generator (Bio-Rad, Hercules, CA, USA) was used prior to DNA amplification with the following conditions: 95 °C for 10 min; 40 cycles of 94 °C for 30 s and 52 °C for 1 min; 98 °C for 10 min. The optimal annealing temperature was selected after performing a temperature gradient assay for each primer/probe set for each gene. DNA amplification was carried out in a C100 Touch™ (Bio-Rad) thermocycler. After the PCR, the QantasoftTM software (Bio-Rad) was used for the analysis, using the RED (rare event detection) option. Samples that did not reach 10,000 events per well were discarded.

### 2.7. Statistical Analyses

Normality of the data was assessed with the Shapiro–Wilk test. The association between TMPRSS4 expression and clinicopathological features of patients was analyzed by Pearson’s chi-square test. Relapse-free survival (RFS) and overall survival (OS), defined as the time from the date of surgery to the date of recurrence or death, respectively, were evaluated with Kaplan–Meier curves, and significant differences among groups were assessed by the log-rank test. For survival analyses, the follow-up period was restricted to 100 months. To evaluate the prognostic value of TMPRSS4, univariable and multivariable Cox proportional hazard analyses were used. Only those variables with *p* ≤ 0.1 in the univariable analysis were included in the multivariable analysis.

To compare levels of *TMPRSS4* and *SHOX2* DNA promoter methylation in plasma and BAL samples from normal individuals vs. patients with NSCLC, the Mann–Whitney U test was used. Receiving operating characteristics (ROC) curves were generated to evaluate the diagnostic ability of the biomarkers. The Youden index was used to find out the optimal cut-off values in the ROC curves and select sensitivity and specificity values. Logistic regression was used to estimate the combined diagnostic potential of both *TMPRSS4* and *SHOX2* in BAL and plasma samples. Statistical analyses were performed with SPSS15.0 (Madrid, Spain), STATA/IC 12.1 (College Station, TX, USA), and GraphPad Prism 5 (San Diego, CA, USA) software. Statistical significance was defined as *p* < 0.05 (*), *p* < 0.01 (**), and *p* < 0.001 (***).

## 3. Results

### 3.1. Prognostic Value of TMPRSS4 Protein Expression in NSCLC

**Figure 1:**
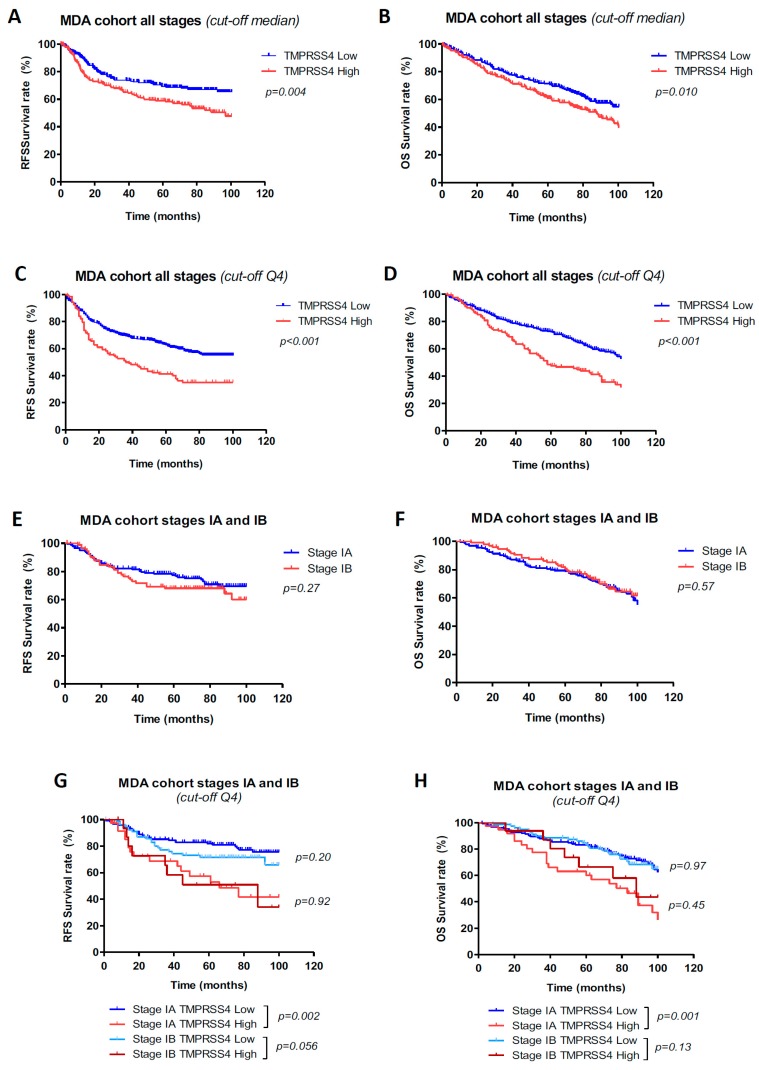
Prognostic value of the Transmembrane protease serine 4 (TMPRSS4) including patients from all stages (I–IV) in the MDA cohort. Relapse-free survival (RFS) (**A**) and overall survival (OS) (**B**) after stratifying the patients based on median protein expression of TMPRSS4. Patients with levels above the median showed worse prognosis. When patients were stratified by quartiles, prognostic differences between both groups were larger: patients with levels above the top 25% (Q4) were associated with lower RFS (*p* < 0.001) (**C**) and OS (*p* < 0.001) (**D**). RFS (**E**) and OS (**F**) comparing stage IA and IB patients. (**G**) RFS analysis in stage IA and IB patients upon stratification by TMPRSS4 protein expression. The top 25% protein expression (Q4) was considered as high level. In the case of stage IA, TMPRSS4 levels in Q4 were significantly associated with lower RFS (*p* = 0.002) as compared to patients within the same stage where TMPRSS4 levels were low. In the case of stage IB, the same tendency was observed for RFS, but results were not statistically different. (**H**) Evaluation of OS rendered similar results to those found for RFS; *n* = 187 stage IA, *n* = 95 stage IB.

TMPRSS4 protein expression was firstly examined in relation to survival. The median H-score was used to categorize patients in high vs. low TMPRSS4 tumor expression. Kaplan–Meier survival curves and log-rank tests considering all NSCLC stages showed that TMPRSS4 levels above the median were significantly associated with both reduced RFS (*p* = 0.004) and OS (*p* = 0.01) in the MDA cohort (489 cases) (Figure 1A,B). Similar results were obtained in the CIMA-CUN cohort (*n* = 95 cases); however, in this case, a tendency toward significance (*p* = 0.12) for RFS and a marginally significant difference (*p* = 0.0499) for OS were observed (Figure S1A,B, Supplementary Materials). The prognostic value of TMPRSS4 expression was maintained in early stages (I–II) for both DFS and OS, in the MDA (*n* = 388) and CIMA-CUN (*n* = 75) cohorts (Figure S1C–F, Supplementary Materials). Relationships between clinicopathological variables and TMPRSS4 expression are shown in Table S5 (Supplementary Materials).

We observed in the TMAs some cases with a very high TMPRSS4 H-score and wondered whether prognosis could be predicted more accurately based on high expression within the top 25% H-score. Therefore, patients were stratified in quartiles, with Q4 representing the one with the highest H-score. Figure 1C,D show an improvement in stratification, where patients with an H-score within Q4 had a much worse outcome (*p* < 0.001 for both RFS and OS) than those included in Q1–Q3. Therefore, this dichotomization was able to better define patients at risk of relapse and death. Such a result was not found in the CIMA-CUN cohort, likely due to limited statistical power. Relationships between clinicopathological variables and TMPRSS4 expression considering Q4 as the cut-off in the MDA cohort are shown in Table S6 (Supplementary Materials).

When the MDA cohort was separated by histologies, in both ADC and SCC, levels over Q4 were also very significantly associated with worse outcome, except for OS in the case of SCC, where only a non-significant trend was observed (Figure S2A–D, Supplementary Materials). Similar results were obtained when the median was considered as a cut-off, but *p*-values were inferior to those found for the top quartile (Figure S2E–H, Supplementary Materials).

To further evaluate the prognostic significance of TMPRSS4 expression, we used univariable and multivariable Cox proportional hazards analysis using Q4 as the cut-off in the MDA cohort. In univariable analysis (Table S7, Supplementary Materials), patients with high TMPRSS4 levels also showed worse RFS (HR, 2.09 (95% CI 1.53–2.87), *p* < 0.001) and OS (HR, 1.82 (95% CI, 1.38–2.41), *p* < 0.001). For the multivariable analysis, we considered variables whose *p*-values were significant or close to significance (*p* ≤ 0.1) in the univariable test. Results shown in Table 2 reveal that TMPRSS4 is an independent prognostic maker of RFS (HR, 1.82, (95% CI, 1.28–2.60), *p* = 0.001) and OS (HR, 1.44, (95% CI, 1.07–1.94), *p* = 0.014).

We then focused on stages IA/IB, as molecular biomarkers might help the stratification of patients at risk of relapse/death and guide clinical management. Clinicopathological characteristics of this subgroup of patients can be found in Table S8 (Supplementary Materials). In the eighth TNM classification of NSCLC, prognosis for stage IB was found not to differ from that of stage IA [3]. In agreement with these results, no differences between both stages were observed for either RFS (*p* = 0.27) or OS (*p* = 0.57) in our cohort of patients (MDA, *n* = 187 stage IA; *n* = 95 stage IB) (Figure 1E,F). However, when considering the protein expression of TMPRSS4 using Q4 as the threshold, we were able to substratify stage IA patients, since those with high TMPRSS4 levels showed a very significantly reduced RFS (*p* = 0.002) and OS (*p* < 0.001) (Figure 1G,H). A similar tendency was observed for stage IB, although statistical differences were not found (Figure 1G,H). Univariable (not shown) and multivariable (Table 3) analysis considering stages IA/B also verified that TMPRSS4 was an independent prognostic factor in this early stage (RFS HR, 2.42 (95% CI, 1.47–3.99), *p* < 0.001; OS HR, 1.99 (95% CI, 1.25–3.16), *p* = 0.004). We also performed this analysis using quartiles in stages II and III–IV and found no significant differences based on TMPRSS4 expression (not shown).

Therefore, we conclude that TMPRSS4 is an independent prognostic marker for NSCLC, especially for very early stages, where it can significantly differentiate patients with a more aggressive disease.

### 3.2. Development of TMPRSS4 and SHOX2 Methylation Assays by ddPCR

Our previous study using pyrosequencing and DNA methylation arrays found significant *TMPRSS4* promoter hypomethylation of certain CpGs in tumors from NSCLC patients as compared to non-malignant lung specimens [7]. Therefore, our next goal was to evaluate whether *TMPRSS4* methylation status could be quantified in liquid biopsy by ddPCR to assess differences between controls and NSCLC patients. To this aim, we set up experimental conditions to evaluate one of these CpGs (cg25116503) within the *TMPRSS4* promoter by ddPCR. In parallel, we developed a ddPCR assay for *SHOX2*, a validated epigenetic diagnostic biomarker for lung cancer. To select the promoter region that differentiates between controls and patients and to design the ddPCR primers, we took into consideration previous reports on SHOX2 methylation in NSCLC that were conducted with other analytical methods [15,17].

**Figure 2:**
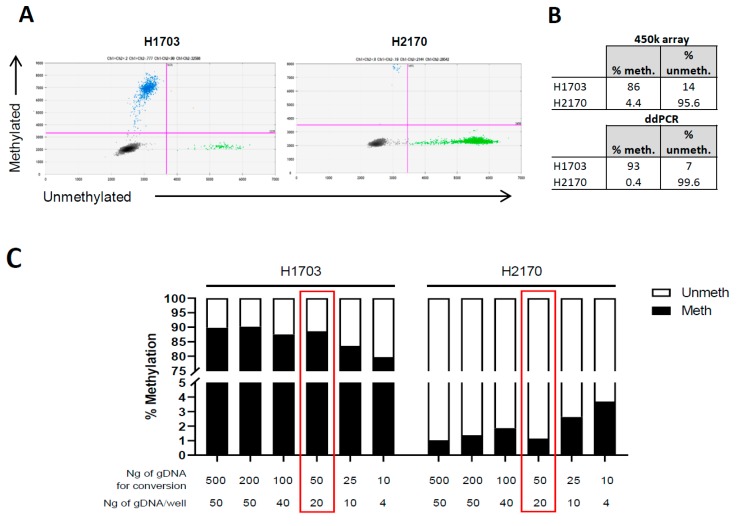

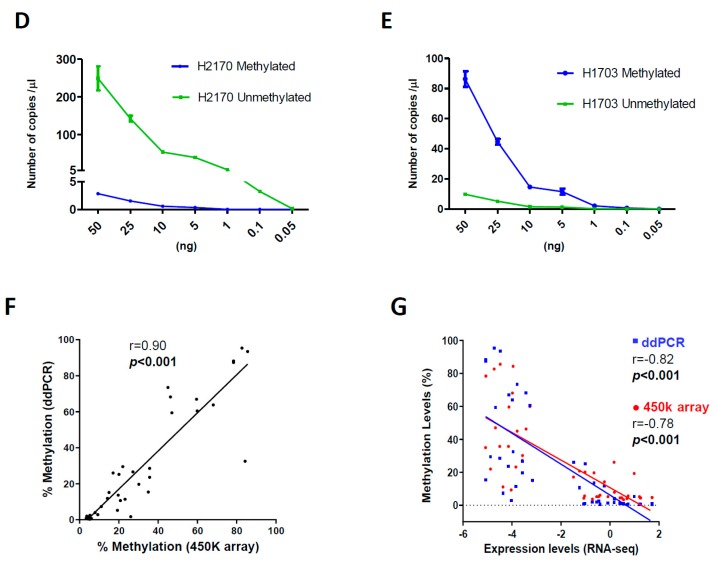
Optimization of conditions for *TMPRSS4* methylation analysis by ddPCR and evaluation of methylation status in lung cancer cell lines. (**A**) Representative two-dimensional (2D) ddPCR plots of number of methylated (blue) and unmethylated (green) events in the cell lines H1703 and H2170. (**B**) Percentages of methylated/unmethylated copies in the cell lines selected as controls (H1703 and H2170). (**C**) Assessment of the amount of DNA for bisulfite conversion and for loading that is necessary for an accurate quantification. The expected percentage of methylated and unmethylated DNA was maintained when at least 50 ng of DNA was converted and 20 ng was loaded. (**D**,**E**) Dilution assays in H2170 and H1703. Upon bisulfite conversion of 50 ng of DNA, serially diluted DNA (indicated in the *X*-axis) was loaded. (**F**) Methylation status of the *TMPRSS4* promoter in a panel of 46 lung cancer cell lines, using 50 ng of DNA as the starting amount and 20 ng for loading. A very significant correlation between ddPCRs and data from the 450k methylation array was found. (**G**) Inverse correlation between methylation status (450k in red and ddPCR in blue) and expression of *TMPRSS4* in the lung cancer cell panel.

As controls for the *TMPRSS4* assay, we selected the following cell lines: H2170, with hypomethylated cg25116503 and high *TMPRSS4* expression, and H1703, with hypermethylated cg25116503 and low *TMPRSS4* expression, based on our previous analysis using the 450k methylation array. We firstly used 500 ng of DNA from these cell lines and quantified the absolute number of methylated and unmethylated copies of cg25116503. Figure 2A shows representative two-dimensional (2D) Quantasoft images in H1703 and H2170, providing evidence that both methylated and unmethylated cg25116503 were accurately amplified in agreement with the expected pattern. The percentage of methylation was highly coincident between the 450K methylation array and the ddPCR (Figure 2B).

Taking into account that DNA concentration in plasma and BAL is low and that bisulfite treatment damages the DNA, we performed ddPCR assays using different starting amounts of DNA for conversion and different amounts of converted DNA to load into the ddPCR wells: 500/50 ng; 200/50 ng; 100/40 ng; 50/20 ng; 25/10 ng; 10/4 ng (Figure 2C). We found that, with the use of an initial DNA amount of 50 ng for conversion and a loading amount of 20 ng, methylation levels were accurately measured, and the expected percentage of methylated/unmethylated copies was maintained (Figure 2C). In parallel, we also did dilution experiments in which, starting from 50 ng and upon conversion, we loaded 50, 25, 10, 5, 1, 0.5, and 0.05 ng per well in each ddPCR reaction. As expected, a concentration-dependent decrease in number of copies/μL was found for both methylated and unmethylated CpGs (Figure 2D,E).

Then, using the combination 50/20 ng DNA, we quantified by ddPCR a panel of 46 lung cancer cell lines in which we previously determined the methylation status of *TMPRSS4* by 450k methylation arrays. As shown in Figure 2F, the methylation status was highly concordant (*r* = 0.90, *p* < 0.001) between both techniques. In agreement with our previous findings [7], *TMPRSS4* methylation levels (determined by either the 450K methylation array or ddPCR) were inversely correlated with *TMPRSS4* messenger RNA (mRNA) levels (*p* < 0.001, Figure 2G).

The same optimization assays were performed for SHOX2 using control cells with high (H2170, 98%), medium (COR-L88, 51%), and low (LXF-289, 37%) methylation levels, based on data from the 450k array (not shown).

**Figure 3:**
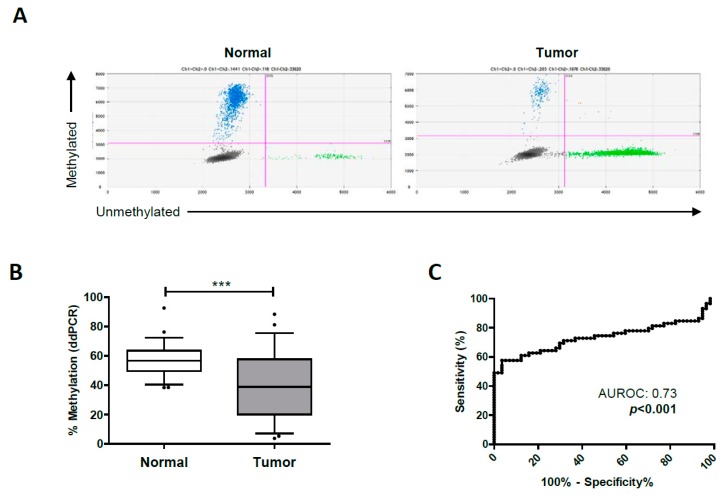
Quantification of the *TMPRSS4* promoter methylation status in normal and tumor samples by ddPCR. (**A**) Representative 2D ddPCR plots of the number of methylated (blue) and unmethylated (green) events in a normal and a malignant sample. (**B**) Tumors from NSCLC patients show a significant hypomethylation in the *TMPRSS4* promoter. (**C**) ROC curve and area under the ROC curve (AUROC) analyzing methylation status of the TMPRSS4 promoter in normal vs. tumors tissue samples. *** *p* < 0.001.

The next step was to quantify the methylation status of the *TMPRSS4* promoter by ddPCR in tumor samples from NSCLC patients, in order to validate our previous results using pyrosequencing and 450k arrays, which showed hypomethylation of cg25116503 in tumors. In the HGUV cohort, comprising 59 stage I–III tumors and their matched non-malignant samples, a significant hypomethylation (*p* < 0.001) of cg25116503 was found in tumors. Representative 2D Quantasoft images for non-malignant and malignant samples are shown in Figure 3A, and average values in patients are shown in Figure 3B. The area under the ROC (AUROC) curve was 0.73 (95% CI 0.63–0.83; *p* < 0.001) (Figure 3C).

### 3.3. Diagnostic Potential of TMPRSS4 and SHOX2 Methylation in Liquid Biopsy Evaluated by ddPCR

**Figure 4:**
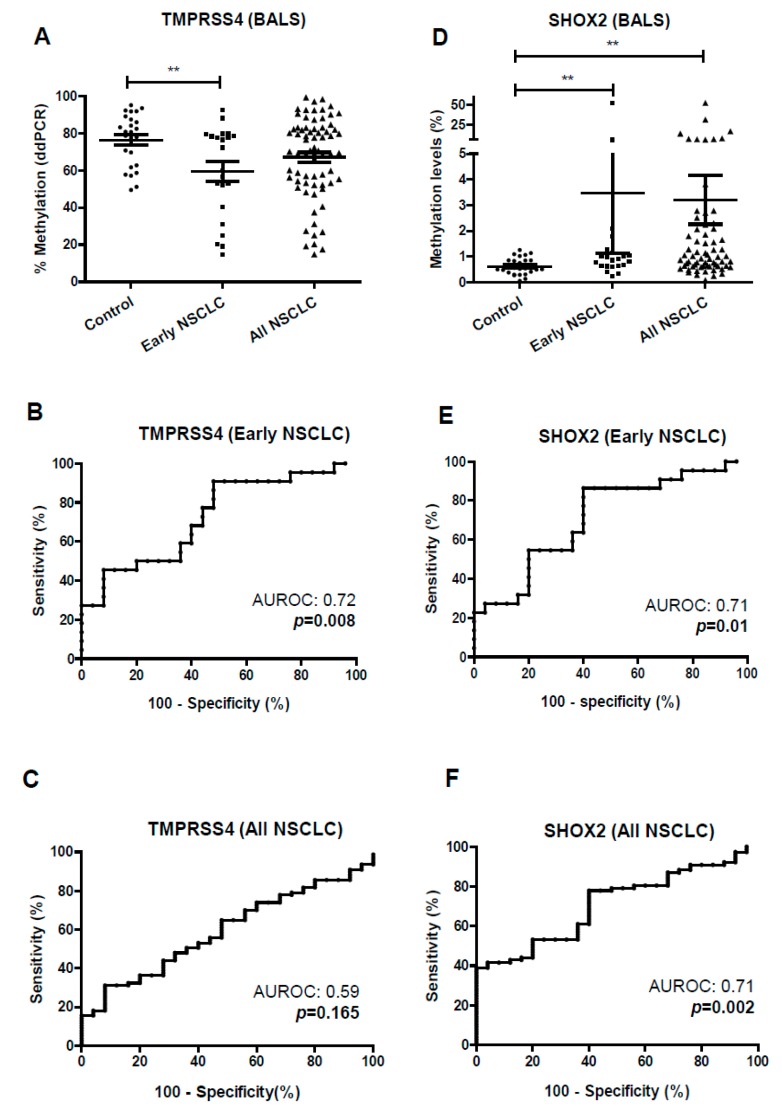
Methylation status of the *TMPRSS4* and *SHOX2* promoters in BAL samples from control individuals and NSCLC patients. Early stages: I–II; all stages: I–IV. (**A**) Significant hypomethylation of *TMPRSS4* (*p* < 0.01) was found in BAL from early-stage NSCLC patients in comparison with controls. (**B**) ROC curve and area under the ROC (AUROC) resulting from the analysis of *TMPRSS4* methylation status in early stages. (**C**) ROC curve and AUROC for *TMPRSS4* methylation status in all stages. (**D**) Significant hypermethylation of *SHOX2* (*p* < 0.01) was observed in BAL for early and late NSCLC stages. (**E**) ROC curve and AUROC for the methylation status of *SHOX2* in early stages. (**F**) The same analysis but considering all stages. ** *p* < 0.01.

To evaluate the performance of the ddPCR in liquid biopsies, we firstly used BAL samples from a cohort of 79 NSCLC patients and 26 controls (tumor-free). Significant hypomethylation (*p* < 0.01) was found for *TMPRSS4* in the case of patients with early stage (I–II) NSCLC in comparison with controls (Figure 4A), with an AUROC of 0.72 ((95% CI, 0.57–0.87), *p* = 0.008) (Figure 4B). The maximum Youden index (maxYouden) was 0.43, with a specificity (SP) and sensitivity (SE) for *TMPRSS4* methylation (*TMPRSS4*^meth^) status in early stage tumors of 91% and 52%, respectively. Considering all stages (I–IV), no significant differences were observed between controls and NSCLC patients (Figure 4A). The value of AUROC in this case was 0.59 ((95% CI, 0.47–0.71), *p* = 0.16) (Figure 4C).

*SHOX2* was significantly hypermethylated in BAL from early-stage NSCLC compared to controls (*p* < 0.01) (Figure 4D) with an AUROC of 0.71 ((95% CI, 0.56–0.86), *p* = 0.01) (Figure 4E). At the maxYouden index of 0.46, SP and SE values were 60% and 86%, respectively. Considering all stages, *SHOX2* methylation status (*SHOX2*^meth^) was also higher in NSCLC patients than in controls (*p* < 0.01) (Figure 4D). The AUROC in this case was 0.71 ((95% CI, 0.60–0.81), *p* = 0.002) (Figure 4F).

A very significant inverse correlation between *TMPRSS4* methylation status and *SHOX2* methylation status was found in the group of early stages (*r* = −0.82; *p* < 0.001) and in all stages (*r* = −0.73; *p* < 0.001) (Figure S3, Supplementary Materials).

**Figure 5:**
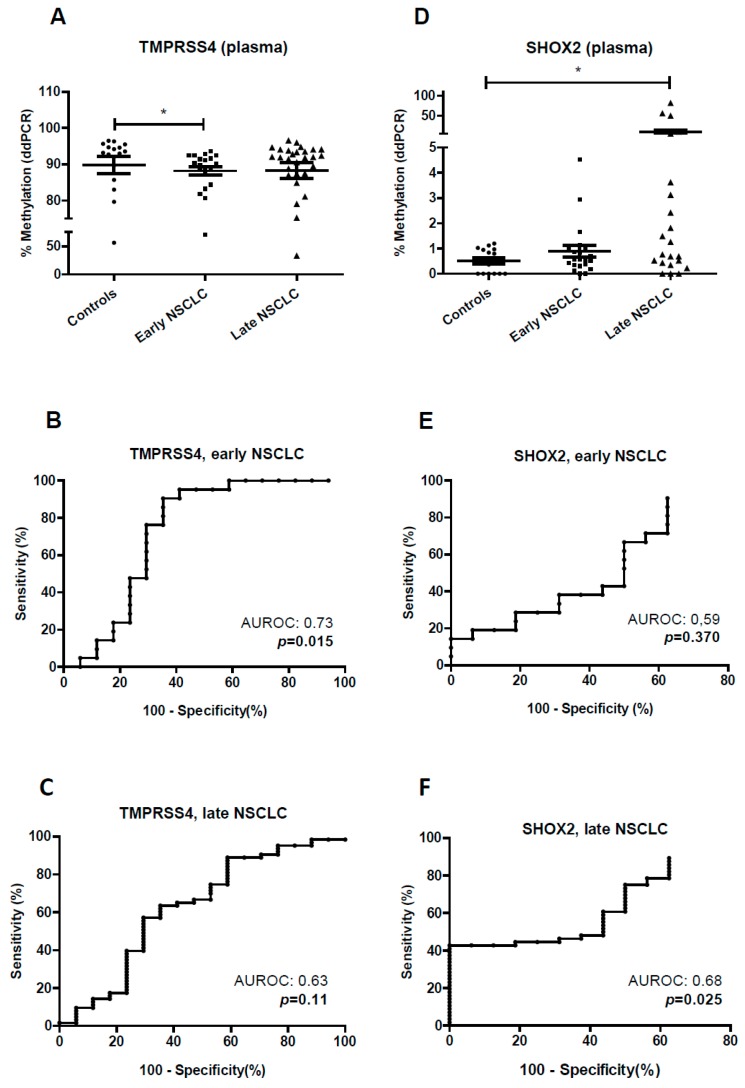
Methylation status of the *TMPRSS4* and *SHOX2* promoter in plasma samples from control individuals and NSCLC patients. Early stages: I–II; late stages: III–IV. (**A**) Significant hypomethylation of the *TMPRSS4* promoter was found in plasma from early stages in comparison with control individuals. (**B**) ROC curve and area under the ROC (AUROC) for *TMPRSS4* methylation status in early stages. (**C**) The same analysis but for late stages. (**D**). Significant hypermethylation of the *SHOX2* promoter was found in plasma only in the case of late stages. (**E**,**F**) ROC curves and AUROC for the *SHOX2* methylation status in the case of early (**E**) or late (**F**) stages. * *p* < 0.05.

We next used plasma from 89 patients with NSCLC and 25 tumor-free individuals (controls) for the study of both *TMPRSS4* and *SHOX2* methylation by ddPCR (1 mL for each gene). In early stages, a significant hypomethylation was found for *TMPRSS4* (*p* < 0.05) (Figure 5A), with an AUROC of 0.73 ((95% CI, 0.54–0.90), *p* = 0.015) (Figure 5B). The maxYouden index was 0.55, and SP and SE were 65% and 90%, respectively. Considering late stages (III–IV), no statistical differences between controls and NSCLC patients were found (Figure 5A). The AUROC in this case was 0.63 ((95% CI, 0.45–0.79), *p* = 0.11) (Figure 5C).

In the case of *SHOX2*, only late-stage NSCLC showed significant hypermethylation with respect to controls (*p* < 0.05) (Figure 5D). The AUROCs for early and late NSCLC were 0.59 ((95% CI, 0.39–0.70), *p* = 0.37) and 0.68 ((95% CI, 0.54–0.80), *p* = 0.025), respectively (Figure 5E,F).

Inverse correlation between *TMPRSS4* methylation and *SHOX2* methylation status was also found for plasma samples in early NSCLC (*r* = −0.56; *p* = 0.007) but not for late NSCLC samples (*r* = 0.01; *p* = 0.9) (Figure S3, Supplementary Materials).

Using logistic regression, we assessed whether a combination of both biomarkers (*TMPRSS4* methylation and *SHOX2* methylation) would increase the diagnostic potential. In the case of BAL, the combined index resulted in a higher AUROC of 0.76 (95% CI, 0.62–0.86), but without statistical differences with each marker alone: *p* = 0.07 when compared to that of *TMPRSS4* methylation and *p* = 0.08 when compared to that of *SHOX2* methylation. In the case of plasma samples, no improvement was found (not shown).

## 4. Discussion

Due to the molecular heterogeneity of NSCLC, some tumors show a particularly malignant phenotype within the same TNM stage and are associated with poor prognosis. Accurate characterization of tumor stage is critical to predict prognosis and to help selecting the optimal treatment options, especially when decisions need to be made in relation to adjuvance in early stages. Therefore, molecular biomarkers that may identify such malignant phenotypes could help in the risk stratification. The latest TNM classification (eighth edition) introduced changes that define prognosis in a more refined way. Our present study about the prognostic role of TMPRSS4 in NSCLC using the eighth TNM classification was based on previous results from our group in a cohort of *n* = 79 patients using the seventh TNM edition, which suggested TMPRSS4 protein expression as a prognostic indicator [7]. Now, we demonstrate here, in a large cohort of patients, that TMPRSS4 protein expression is an independent prognostic indicator, especially for very early stages, which can significantly differentiate individuals with a more aggressive disease.

In agreement with results from the eighth TNM classification of NSCLC [3], our study shows that prognosis of stages IA and IB does not differ. Although adjuvant chemotherapy is routinely performed in stages II–III, its use in stage IA is detrimental. Regarding stage IB, there is still controversy, as some studies described beneficial effects [22] while others found the opposite results [23,24,25]. Therefore, molecular biomarkers that may distinguish patients at high risk of recurrence within these early stages could contribute to clinical decisions, although such biomarkers are currently lacking. Our study shows that prognosis in patients with stage IA TMPRSS4^high^ is significantly worse than that of patients with stage IA TMPRSS4^low^ and overlaps with that found in patients with stage IB TMPRSS4^low^. Thus, high expression of TMPRSS4 in stage IA may uncover a subgroup of patients with a more malignant phenotype. In the future, upon extended implementation of low-dose CT-based screening programs, the proportion and total numbers of stage I–II NSCLC cases diagnosed will increase. Thus, the need for new and robust tools for indeterminate nodule characterization and for early tumor prognostic classification will be more compelling [26].

As a hypothesis, we can speculate that the malignant phenotype in TMPRSS4^high^ tumors would be related to the acquisition of epithelial-to-mesenchymal transition (EMT) and cancer stem cell (CSC) characteristics early during carcinogenesis, which could induce their escape from the primary tumor. We previously described that TMPRSS4 overexpression promotes metastatic dissemination and resistance to chemotherapy in experimental models [5,27]. In agreement with these results, downregulation of TMPRSS4 highly sensitizes lung cancer cells to chemotherapy, including cisplatin and paclitaxel [5]. Therefore, TMPRSS4 deserves further consideration as a protumorigenic target and a prognostic biomarker in early stage NSCLC.

One clinically unmet need is the identification of biomarkers to follow up early-stage lung cancer patients who, after undergoing surgery, will relapse. In line with the prognostic role of TMPRSS4, we sought to develop a non-invasive method using highly sensitive techniques to differentiate between controls and NSCLC patients. Because TMPRSS4 expression in NSCLC is associated with hypomethylation of the DNA promoter and reduced methylation is related to poor outcome [7], we decided to investigate whether *TMPRSS4* methylation status could constitute a biomarker of malignancy in liquid biopsy. Epigenetic changes occur soon during tumor development and, thus, they give promise as diagnostic biomarkers for early stages [28]. Moreover, DNA methylation is a covalent and stable modification that can be detected in circulating free DNA. In the clinic, the most relevant epigenetic marker is O-6-Methylguanine-DNA Methyltransferase (*MGMT*) as a predictor of response to chemotherapy in glioblastoma [29]. In the case of lung cancer, several studies showed the diagnostic potential of *SHOX2*, which was commercialized (www.epigenomics.com).

Conventional methylation-specific PCR methods are commonly used to quantify the DNA methylation status of different genes, but these methods show some shortcomings for their clinical use. Firstly, they require internal controls for normalization, and sensitivity is in many cases insufficient when analyzing low amounts of DNA (as is the case for plasma and BAL). However, ddPCR does not depend on the presence of internal calibration curves and provides an absolute copy number. Moreover, ddPCR can quantify low-abundance nucleic acids with much higher sensitivity than conventional PCR and, therefore, it is a more suitable method to evaluate DNA methylation changes in liquid biopsy.

In our study, we proved that methylation status of *TMPRSS4* and *SHOX2* can be accurately quantified by ddPCR in both plasma and BAL from NSCLC patients and healthy individuals. The AUROC curves for *TMPRSS4* in plasma and BAL from early-stage (I–II) NSCLC and controls were 0.73 and 0.72, respectively. These data are similar to the values we found for tumors (AUROC = 0.73) and suggest that DNA methylation changes occurring within the tumor are also reflected in liquid biopsies. It is worth noting that the methylation status of *TMPRSS4* was inversely correlated with the methylation status of *SHOX2* in BAL but not in plasma. In our study, hypermethylation of *SHOX2* showed lower diagnostic power than the one reported by other studies for plasma or BAL: AUROC curves within the range of 0.78 to 0.91 [15,16,17]. This could be related to the large number of patients used in those studies or due to methodological differences.

Because of tumor heterogeneity, it is likely that combination of different epigenetic markers improves the diagnostic capability, although this was not the case in our study when combining *TMPRSS4* and *SHOX2*. A report combining *SHOX2* and *PTEGR4* (Prostaglandin E receptor 4) in plasma using several cohorts of patients obtained AUROC curves between 0.88 and 0.98 [30]. An epigenetic classifier including four genes (*HOXA9*, *RASSF1A*, *SOX17*, and *TAC1*) that was evaluated in sputum by ddPCR reached an AUROC of 0.92 for lung cancer detection [31]. Another classifier that included genes *BCAT1*, *CDO1*, *TRIM58*, and *ZNF177* was analyzed using a logistic regression model in bronchial fluids, with a combined AUROC value of 0.91 [32].

Cytological examination of BAL is an important diagnostic method in NSCLC. Nonetheless, the sensitivity of this method is modest, with values within 45–50% reported in many studies (reviewed by Reference [33]). Some papers showed that changes in DNA methylation of different genes have a better diagnostic potential than cytology or can be used in combination with cytological analysis to increase sensitivity, although those results are in need of validation. For example, Isle et al. [34] showed that *SHOX2* hypermethylation aids cytology in NSCLC diagnosis. Also, the four-gene classifier mentioned above [32] improved diagnosis by cytological examination. Therefore, analysis of aberrant methylation in ct free DNA/exfoliated tumor cells found in BAL may help to increase the sensitivity of cytology. A future prospective study combining *TMPRSS4* methylation status and cytology could validate the role of this potential epigenetic biomarker in BAL samples. 

Determination of methylation status of *TMPRSS4* (*TMPRSS4*^meth^ status) in blood may be useful in a clinical scenario where a surgically treated patient is at risk based on both stage and high *TMPRSS4* tumor expression. The subsequent liquid biopsies based on the ddPCR measurement of *TMPRSS4*^meth^ status in blood could be used as an auxiliary test to follow the patient’s evolution, eventually suggesting the presence of a relapsed tumor. Further studies carried out in intended-to-use cohorts (in screening or prognostic settings) may clarify whether this new liquid biopsy technology is clinically validated in diagnostic or prognostic trials.

## 5. Conclusions

We conclude from our study that TMPRSS4 is an independent prognostic indicator of reduced RFS and OS in early NSCLC, and that *TMPRSS4*^meth^ status differentiates between tumor-free individuals and those with NSCLC. Our data support the use of TMPRSS4 as an indicator of malignancy in early-stage NSCLC.

## Figures and Tables

**Table 1:** Cohorts of patients used to study the prognostic value of (TMPRSS4) by immunohistochemistry.

		MDA Cohort (*n* = 489)	CIMA-CUN Cohort (*n* = 95)
		*n* (%)	*n* (%)
Age	<65	180 (36.8)	47 (49.5)
	≥65	309 (63.2)	48 (50.5)
Gender	Female	260 (53.2)	13 (13.7)
	Male	229 (46.8)	82 (86.3)
Smoking habits	Current	167 (34.2)	21 (22.1)
	Former	255 (52.1)	66 (69.5)
	Never	67 (13.7)	8 (8.4)
Histology	ADC	299 (61.1)	45 (47.4)
	SCC	171 (35.0)	46 (48.4)
	Other	19 (3.9)	4 (4.2)
Stage (8th edition)	I	282 (57.7)	44 (46.3)
	II	106 (21.7)	31 (32.6)
	III	92 (18.8)	18 (18.9)
	IV	9 (1.8)	2 (2.1)
Adjuvant therapy	No	317 (64.8)	54 (56.8)
	Yes	119 (24.3)	41 (43.2)
	NA *	53 (10.8)	

* Not available; ADC: adenocarcinoma; SCC: squamous cell carcinoma.

**Table 2:** Multivariable Cox proportional hazards analysis of TMPRSS4 protein expression and relapse-free survival (RFS) or overall survival (OS) in the MDA cohort. Dichotomization of TMPRSS4 expression was based on quartiles (considering (the top 25%, Q4) as high). The prognostic value of TMPRSS4 for both RFS and OS in this early stage is independent of other variables. Only variables whose *p*-value was ≤0.1 were considered.

	MDA Cohort (*n* = 489)						
		RFS			OS		
		HR	95% CI	*p*	HR	95% CI	*p*
Age	<65				1		
	≥65				1.83	(1.35–2.47)	**<0.001**
Gender	Female				1		
	Male				0.83	(0.63–1.10)	0.211
Smoking habits	Never	1			1		
	Former	1.66	(0.88–3.15)	0.115	1.22	(0.74–2.02)	0.416
	Current	1.99	(1.02–3.87)	**0.041**	1.62	(0.96–2.73)	0.066
Histology	ADC				1		
	SCC				1.4	(1.05–1.08)	**0.02**
	Other				1.35	(0.66–2.78)	0.406
Stage (8th edition)	I	1			1		
	II	1.99	(1.29–3.08)	**0.002**	2.15	(1.55–2.97)	**<0.001**
	III–IV	2.73	(1.70–4.37)	**<0.001**	2.68	(1.92–3.74)	**<0.001**
Adjuvant therapy	No	1					
	Yes	0.72	(0.47–1.09)	0.122			
TMPRSS4	Low	1			1		
	High	1.82	(1.28–2.60)	**0.001**	1.44	(1.07–1.94)	**0.014**

RFS: Relapse-free survival; OS: overall survival.

**Table 3:** Multivariable Cox proportional hazards analysis of TMPRSS4 protein expression and RFS or OS in stage IA/IB patients from the MDA cohort. Dichotomization of TMPRSS4 expression was based on quartiles (considering the top 25%, Q4 as high). The prognostic value of TMPRSS4 for both RFS and OS in this early stage is independent of other variables. Only variables whose *p*-value was ≤0.1 in the univariable analysis were considered.

	MDA Cohort (*n* = 283)						
		RFS			OS		
		HR	95% CI	*p*	HR	95% CI	*p*
Age	<65				1		0.292
	≥65				1.28	(0.80–2.04)	
Gender	Female				1		0.076
	Male				0.67	(0.43–1.04)	
Smoking habits	Current	1			1		
	Former	1.73	(0.76–3.93)	0.183	1.37	(0.69–2.73)	0.352
	Never	1.93	(0.82–4.50)	0.127	1.24	(0.59–2.61)	0.56
Histology	ADC				1		
	SCC				1.53	(0.97–2.39)	0.062
	Other				2.73	(1.16–6.43)	**0.021**
Stage (8th edition)	IA	1					
	IB	1.27	(0.79–2.02)	0.31			
TMPRSS4	Low	1			1		
	High	2.42	(1.47–3.99)	**<0.001**	1.99	(1.25-3.16)	**0.004**

## Supplementary Materials

The following are available online at https://www.mdpi.com/2077-0383/8/12/2134/s1. Figure S1: Prognostic value of TMPRSS4 including patients from all stages (I–IV) and early stages (I–II) in the CIMA-CUN cohort, and early stages (I–II) in the MDA cohort. RFS (**A**) and OS (**B**) in patients from CIMA-CUN stratified by the median value of TMPRSS4 protein expression. Differences between good/bad prognosis was clearly appreciated in stage I–II patients: *p* = 0.006 for RFS (**C**) and *p* < 0.001 for OS (**D**). RFS (**E**) and OS (**F**) for stages I–II from the MDA cohort stratified by the median.; Figure S2: Prognostic value of TMPRSS4 from the MDA cohort in adenocarcinomas (ADC) or squamous cell carcinomas (SCC), considering all stages. In (**A**–**D**), the top quartile (Q4) was considered as cut-off value. (**A**) RFS in SCC; (**B**) RFS in ADC; (**C**) OS in SCC; (**D**) OS in ADC. In (**E**–**H**), the median was also evaluated as cut-off. (**E**) RFS in SCC; (**F**) RFS in ADC; (**G**) OS in SCC; (**H**) OS in ADC. Separation of the patients in high/low risk and *p*-values of the differences were improved when quartiles were considered for the stratification.; Figure S3: Inverse correlation between *TMPRSS4* methylation status and *SHOX2* methylation status in BAL (**A**,**B**) and plasma (**C**,**D**).; Table S1: Cohort of patients from Hospital General Universitario de Valencia (HGUV) used to study the methylation status of *TMPRSS4* and *SHOX2* in tumors and matched non-malignant lung samples by ddPCR; Table S2: Cohort of patients used to quantify methylation status of the *TMPRSS4* and *SHOX2* promoters in bronchoalveolar lavages (BAL) from NSCLC patients; Table S3: Cohort of patients used to quantify methylation status of the *TMPRSS4* and *SHOX2* promoters in plasmas from NSCLC patients; Table S4: Sequence of primers and probes for ddPCR analysis; Table S5: Relationship between TMPRSS4 protein levels and clinicopathological characteristics of the patients from the MDA and CIMA-CUN cohorts; Table S6: Relationship between TMPRSS4 protein levels and clinicopathological characteristics of the patients from the MDA cohort using the top quartile (Q4) as the cut-off value; Table S7: Univariable Cox proportional hazards analysis of TMPRSS4 protein expression and RFS or OS in the MDA cohort; Table S8: Clinicopathological characteristics of stage IA/IB NSCLC patients from the MDA cohort that were used to study the prognostic value of TMPRSS4.

## References

[1] Siegel R.L., Miller K.D., Jemal A. (2019). Cancer statistics, 2019. CA Cancer J. Clin..

[2] Uramoto H., Tanaka F. (2014). Recurrence after surgery in patients with NSCLC. Transl. Lung Cancer Res..

[3] Goldstraw P., Chansky K., Crowley J., Rami-Porta R., Asamura H., Eberhardt W.E.E., Nicholson A.G., Groome P., Mitchell A., Bolejack V. (2016). The IASLC Lung Cancer Staging Project: Proposals for Revision of the TNM Stage Groupings in the Forthcoming (Eighth) Edition of the TNM Classification for Lung Cancer. J. Thorac. Oncol..

[4] Burotto M., Thomas A., Subramaniam D., Giaccone G., Rajan A. (2014). Biomarkers in Early-Stage Non–Small-Cell Lung Cancer: Current Concepts and Future Directions. J. Thorac. Oncol..

[5] Exposito F., Villalba M., Redrado M., de Aberasturi A.L., Cirauqui C., Redin E., Guruceaga E., de Andrea C., Vicent S., Ajona D. (2019). Targeting of TMPRSS4 sensitizes lung cancer cells to chemotherapy by impairing the proliferation machinery. Cancer Lett..

[6] Wang C.-H., Guo Z.-Y., Chen Z.-T., Zhi X.-T., Li D.-K., Dong Z.-R., Chen Z.Q., Hu S.Y., Li T. (2015). TMPRSS4 facilitates epithelial-mesenchymal transition of hepatocellular carcinoma and is a predictive marker for poor prognosis of patients after curative resection. Sci. Rep..

[7] Villalba M., Diaz-Lagares A., Redrado M., de Aberasturi A.L., Segura V., Bodegas M.E., Pajares M.J., Pio R., Freire J., Gomez-Roman J. (2016). Epigenetic alterations leading to TMPRSS4 promoter hypomethylation and protein overexpression predict poor prognosis in squamous lung cancer patients. Oncotarget.

[8] Cheng Y., Wang K., Geng L., Sun J., Xu W., Liu D., Gong S., Zhu Y. (2019). Identification of candidate diagnostic and prognostic biomarkers for pancreatic carcinoma. EBioMedicine.

[9] Liang B., Wu M., Bu Y., Zhao A., Xie F. (2013). Prognostic value of TMPRSS4 expression in patients with breast cancer. Med. Oncol..

[10] Cheng D., Kong H., Li Y. (2013). TMPRSS4 as a Poor Prognostic Factor for Triple-Negative Breast Cancer. Int. J. Mol. Sci..

[11] Liu G.T., Shen C., Ren X.H., Yang L., Yu Y.M., Xiu Y.X., Li R.H., Jiang L., Zhang C.L., Li Y.W. (2017). Relationship between transmembrane serine protease expression and prognosis of esophageal squamous cell carcinoma. J. Biol. Regul. Homeost. Agents.

[12] Kim S., Ko D., Lee Y., Jang S., Lee Y., Lee I.Y., Kim S. (2019). Anti-cancer activity of the novel 2-hydroxydiarylamide derivatives IMD-0354 and KRT1853 through suppression of cancer cell invasion, proliferation, and survival mediated by TMPRSS4. Sci. Rep..

[13] Pisapia P., Malapelle U., Troncone G. (2018). Liquid Biopsy and Lung Cancer. Acta Cytol..

[14] Alegre E., Fusco J.P., Restituto P., Salas-Benito D., Rodríguez-Ruiz M.E., Andueza M.P., Pajares M.J., Patiño-García A., Pio R., Lozano M.D. (2016). Total and mutated EGFR quantification in cell-free DNA from non-small cell lung cancer patients detects tumor heterogeneity and presents prognostic value. Tumor Biol..

[15] Kneip C., Schmidt B., Seegebarth A., Weickmann S., Fleischhacker M., Liebenberg V., Field J.K., Dietrich D. (2011). SHOX2 DNA Methylation Is a Biomarker for the Diagnosis of Lung Cancer in Plasma. J. Thorac. Oncol..

[16] Konecny M., Markus J., Waczulikova I., Dolesova L., Kozlova R., Repiska V., Novosadova H., Majer I. (2016). The value of SHOX2 methylation test in peripheral blood samples used for the differential diagnosis of lung cancer and other lung disorders. Neoplasma.

[17] Schmidt B., Beyer J., Dietrich D., Bork I., Liebenberg V., Fleischhacker M. (2015). Quantification of cell-free mSHOX2 Plasma DNA for therapy monitoring in advanced stage non-small cell (NSCLC) and small-cell lung cancer (SCLC) patients. PLoS ONE Public Libr. Sci..

[18] Schmidt B., Liebenberg V., Dietrich D., Schlegel T., Kneip C., Seegebarth A., Field J.K., Dietrich D. (2010). SHOX2 DNA Methylation is a Biomarker for the diagnosis of lung cancer based on bronchial aspirates. BMC Cancer.

[19] Altman D.G., McShane L.M., Sauerbrei W., Taube S.E. (2012). Reporting Recommendations for Tumor Marker Prognostic Studies (REMARK): Explanation and Elaboration. PLoS Med..

[20] Villalba M., Lopez L., Redrado M., Ruiz T., de Aberasturi A.L., de la Roja N., Garcia D., Exposito F., de Andrea C., Alvarez-Fernandez E. (2017). Development of biological tools to assess the role of TMPRSS4 and identification of novel tumor types with high expression of this prometastatic protein. Histol. Histopathol..

[21] Martínez-Terroba E., Behrens C., de Miguel F.J., Agorreta J., Monsó E., Millares L., Sainz C., Mesa-Guzman M., Pérez-Gracia J.L., Lozano M.D. (2018). A novel protein-based prognostic signature improves risk stratification to guide clinical management in early-stage lung adenocarcinoma patients. J. Pathol..

[22] Roselli M., Mariotti S., Ferroni P., Laudisi A., Mineo D., Pompeo E., Mineo T.C. (2006). Postsurgical chemotherapy in stage IB nonsmall cell lung cancer: Long-term survival in a randomized study. Int. J. Cancer.

[23] Park J., Lee C., Lee H., Baek H., Zo J., Shim Y. (2005). Postoperative adjuvant chemotherapy for stage I non-small cell lung cancer. Eur. J. Cardio Thorac. Surg..

[24] Park H.J., Park H.S., Cha Y.J., Lee S., Jeung H.-C., Cho J.Y., Kim H.J., Byun M.K. (2018). Efficacy of adjuvant chemotherapy for completely resected stage IB non-small cell lung cancer: A retrospective study. J. Thorac. Dis..

[25] Wang J., Wu N., Lv C., Yan S., Yang Y. (2019). Should patients with stage IB non-small cell lung cancer receive adjuvant chemotherapy? A comparison of survival between the 8th and 7th editions of the AJCC TNM staging system for stage IB patients. J. Cancer Res. Clin. Oncol..

[26] Seijo L.M., Peled N., Ajona D., Boeri M., Field J.K., Sozzi G., Pio R., Zulueta J.J., Spira A., Massion P.P. (2019). Biomarkers in Lung Cancer Screening: Achievements, Promises, and Challenges. J. Thorac. Oncol..

[27] De Aberasturi A.L., Redrado M., Villalba M., Larzabal L., Pajares M.J., Garcia J., Evans S.R., Garcia-Ros D., Bodegas M.E., Lopez L. (2016). TMPRSS4 induces cancer stem cell-like properties in lung cancer cells and correlates with ALDH expression in NSCLC patients. Cancer Lett..

[28] Mari-Alexandre J., Diaz-Lagares A., Villalba M., Juan O., Crujeiras A.B., Calvo A., Sandoval J. (2017). Translating cancer epigenomics into the clinic: Focus on lung cancer. Transl. Res..

[29] Martinez R., Esteller M. (2010). The DNA methylome of glioblastoma multiforme. Neurobiol. Dis..

[30] Weiss G., Schlegel A., Kottwitz D., König T., Tetzner R. (2017). Validation of the SHOX2/PTGER4 DNA Methylation Marker Panel for Plasma-Based Discrimination between Patients with Malignant and Nonmalignant Lung Disease. J. Thorac. Oncol..

[31] Su Y., Fang H., Bin Jiang F. (2018). An epigenetic classifier for early stage lung cancer. Clin. Epigenet. BioMed Cent..

[32] Diaz-Lagares A., Mendez-Gonzalez J., Hervas D., Saigi M., Pajares M.J., Garcia D., Crujerias A.B., Pio R., Montuenga L.M., Zulueta J. (2016). A Novel Epigenetic Signature for Early Diagnosis in Lung Cancer. Clin. Cancer Res..

[33] Schreiber G., McCrory D.C. (2003). Performance characteristics of different modalities for diagnosis of suspected lung cancer: Summary of published evidence. Chest.

[34] Ilse P., Biesterfeld S., Pomjanski N., Wrobel C., Schramm M. (2014). Analysis of SHOX2 methylation as an aid to cytology in lung cancer diagnosis. Cancer Genom. Proteom..

