# Supplementary material for: TMPRSS4: A Novel Tumor Prognostic Indicator for the Stratification of Stage IA Tumors and a Liquid Biopsy Biomarker for NSCLC Patients
Source: J Clin Med. 2019 Dec 3;8(12):2134. doi: 10.3390/jcm8122134 (PMC6947244; doi:10.3390/jcm8122134)
Supplement: Supplementary file 1 [file jcm-08-02134-s001.pdf]

Journal of
Clinical Medicine

MDPI

Supplementary Materials

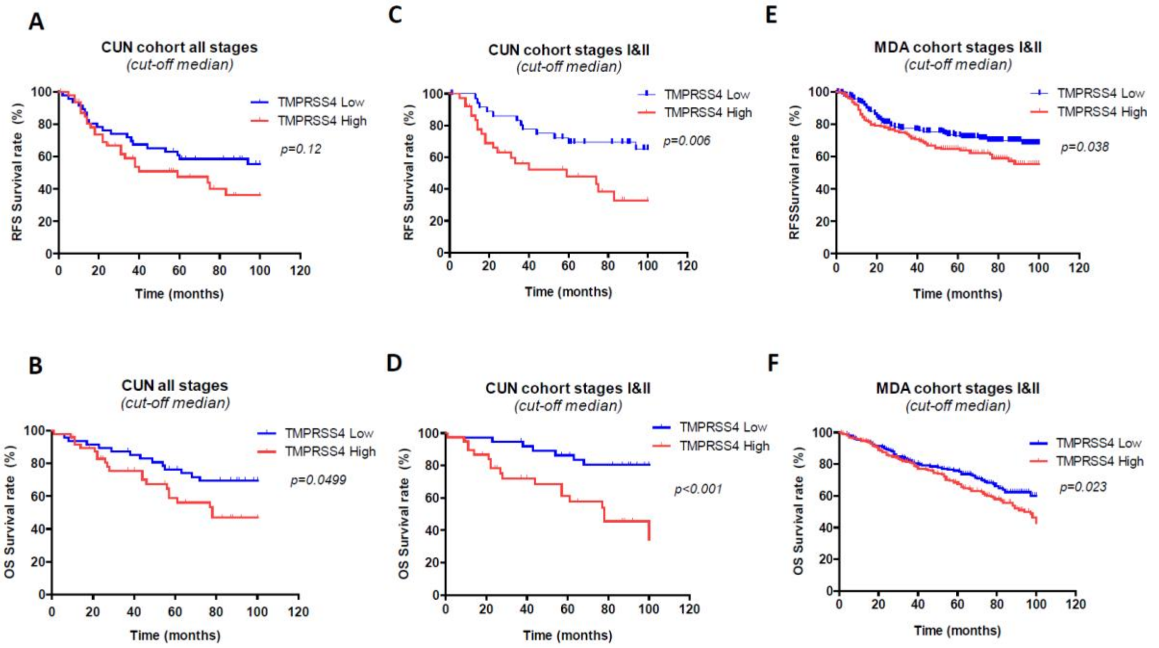

Figure S1. Prognostic value of TMPRSS4 including patients from all stages (I–IV) and early stages (I–II) in the CIMA-CUN cohort, and early stages (I–II) in the MDA cohort. RFS (A) and OS (B) in patients from CIMA-CUN stratified by the median value of TMPRSS4 protein expression. Differences between good/bad prognosis was clearly appreciated in stage I-II patients: p=0.006 for RFS (C) and p<0.001 for OS (D). RFS (E) and OS (F) for stages I-II from the MDA cohort stratified by the median.

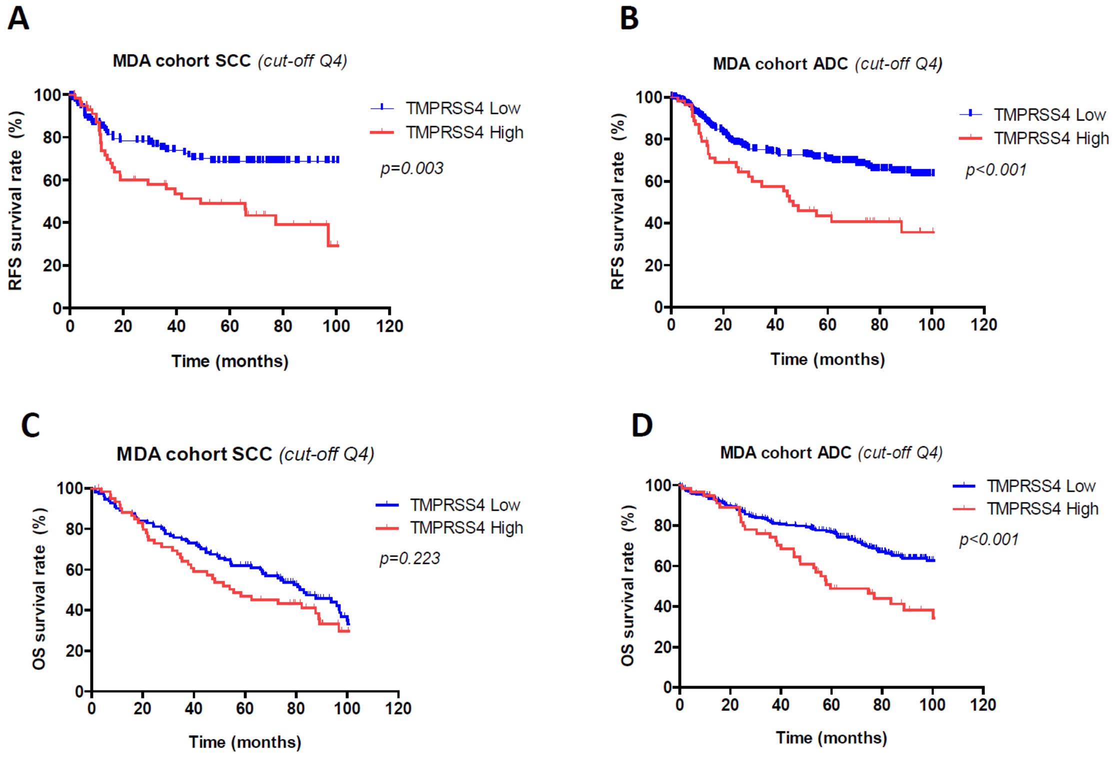

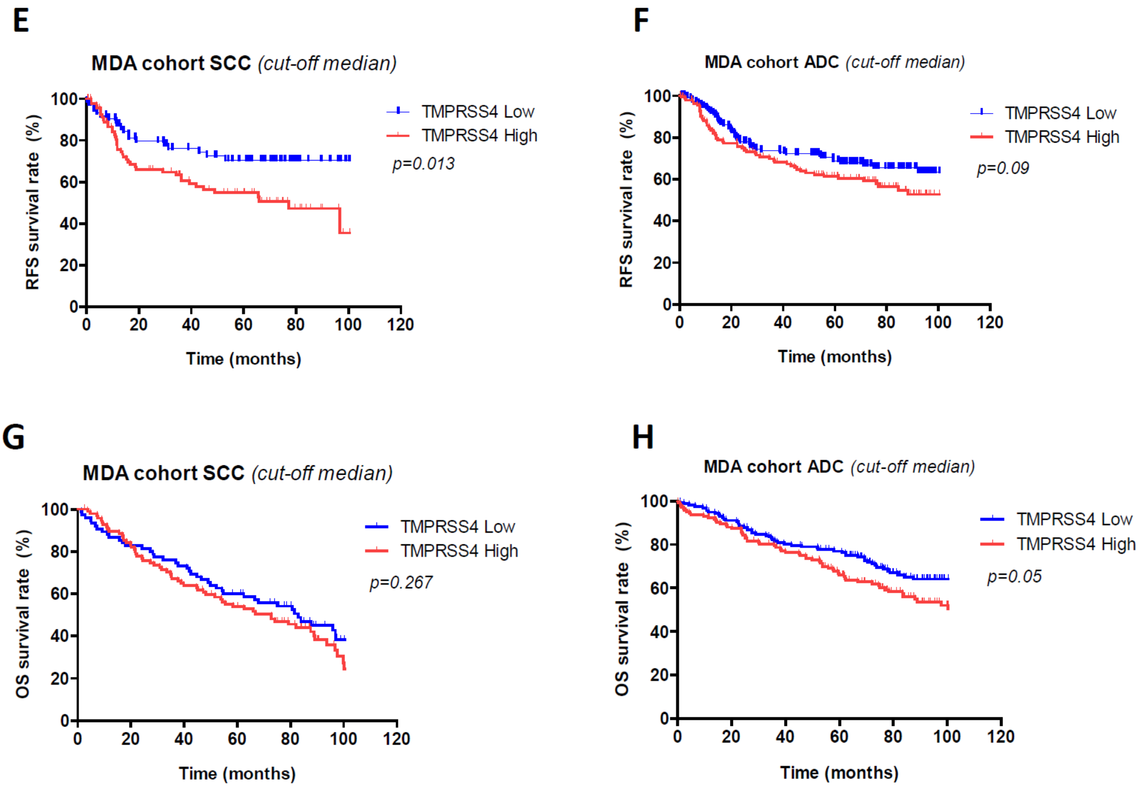

Figure S2. Prognostic value of TMPRSS4 from the MDA cohort in adenocarcinomas (ADC) or squamous cell carcinomas (SCC), considering all stages. In A-D, the top quartile (Q4) was considered as cut-off value. (A) RFS in SCC; (B) RFS in ADC; (C) OS in SCC; (D) OS in ADC. In E-H, the median was also evaluated as cut-off. (E) RFS in SCC; (F) RFS in ADC; (G) OS in SCC; (H) OS in ADC. Separation of the patients in high/low risk and p-values of the differences were improved when quartiles were considered for the stratification.

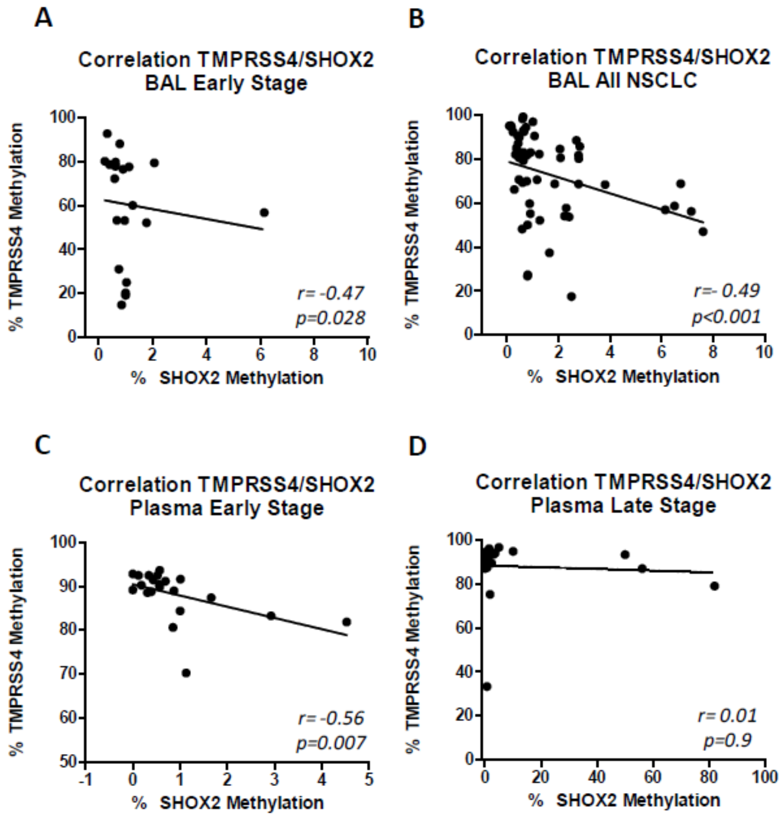

Figure S3. Inverse correlation between *TMPRSS4* methylation status and *SHOX2* methylation status in BAL (**A**,**B**) and plasma (**C**,**D**).

Table S1. Cohort of patients from Hospital General Universitario de Valencia (HGUV) used to study the methylation status of TMPRSS4 and SHOX2 in tumors and matched non-malignant lung samples by ddPCR.

**HGUV cohort (n=59)**	
**Age**	**n (%)**
<65	27 (45.8)
≥65	32 (54.2)
**Gender**	
Female	1 (1.7)
Male	58 (98.3)
**Smoking habit**	
Current	30 (50.8)
Former	27 (45.8)
Never	2 (3.4)
**Histology**	
SCC	57 (96.6)
Others	2 (3.4)
**Stage**	
I	27 (45.8)
II	14 (23.7)
III	18 (30.5)
**Adjuvant therapy**	
No	32 (54.2)
Yes	22 (37.3)
NA *	5 (8.5)

*Not available; SCC: squamous cell carcinoma

Table S2. Cohort of patients used to quantify methylation status of the *TMPRSS4* and *SHOX2* promoters in bronchoalveolar lavages (BAL) from NSCLC patients.

BAL cohort (n=79)	
**Age**	**n (%)**
<65	51 (64.6)
≥65	28 (35.4)
**Gender**	
Female	14 (17.7)
Male	65 (82.3)
**Smoking status**	
Current	21 (26.6)
Former	43 (54.4)
Never	14 (17.7)
NA *	1 (1.3)
**Histology**	
ADC	22 (27.8)
SCC	38 (48.1)
Others	16 (20.3)
NA *	3 (3.8)
**Stage**	
I	15 (19.0)
II	8 (10.0)
III	20 (25.4)
IV	18 (22.8)
NA *	18 (22.8)

*Not available; ADC: adenocarcinoma; SCC: squamous cell carcinoma

Table S3. Cohort of patients used to quantify methylation status of the *TMPRSS4* and *SHOX2* promoters in plasmas from NSCLC patients.

Plasmas cohort (n=89)	
**Age**	**n (%)**
<65	56 (62.9)
≥65	33 (37.1)
**Gender**	
Female	23 (25.8)
Men	66 (74.2)
**Smoking status**	
Current	26 (29.3)
Former	48 (53.9)
Never	14 (15.7)
NA*	1 (1.1)
**Histology**	
ADC	51 (57.3)
SCC	38 (42.7)
**Stage**	
I	8 (9.0)
II	8 (9.0)
III	19 (21.4)
IV	52 (58.4)
NA*	2 (2.2)

*Not available; ADC: adenocarcinoma; SCC: squamous cell carcinoma

Table S4. Sequence of primers and probes for ddPCR analysis.

Gene	Sequence (5' - 3') bisulfite-treated DNA		Sequence (5' - 3') untreated DNA	
TMPRSS4	**Fw:**	GTTGGGTGGAAGTTATTTAG	**Fw:**	CCTGTTCTGAGGCCGAAG
	**Rev:**	ACTAAAACAACACCTAAAATC	**Rev:**	GTGTCTTGTAACCTGACACC
	**Probe Met (FAM):**	TGAGGT**CG**AAGGTTTTATG	**Probe (FAM):**	ACTTCAGGTGTTGTTCCAGCC
	**Probe non-Met (HEX):**	TGAGGT**TG**AAGGTTTTATG		
SHOX2	**Fw:**	AGGTTTTTTGGATAGTTAGG	**Fw:**	AGGCGGAAAACCAGCCTC
	**Rev:**	ACCCTTTAAACAACCAAC	**Rev:**	TGCGTTTCCACGAGGGAG
	**Probe Met (FAM):**	A**CG**AGTATAGG**CG**TTTA**CG**T	**Probe (FAM):**	CCGCTCTTTCTGTTCTCTCTTCGC
	**Probe non-Met (HEX):**	TA**TG**AGTATAGG**TG**TTTA**TG**TTAT		

Table S5. Relationship between TMPRSS4 protein levels and clinicopathological characteristics of the patients from the MDA and CIMA-CUN cohorts. The median was used as cut-off value to define high vs. low expression.

**MDA Cohort (n=489)**				**CIMA-CUN Cohort (n=95)**			
	**TMP Low**	**TMP High**	***p***		**TMP Low**	**TMP High**	***p***
**Age**	***n* (%)**	***n* (%)**		**Age**	***n* (%)**	***n* (%)**	
< 65	94 (52.2)	86 (47.8)	0.356	< 65	27 (57.4)	20 (42.6)	0.124
≥65	148 (47.8)	161 (52.1)		≥65	20 (41.7)	28 (58.3)	
**Gender**				**Gender**			
Female	111 (48.5)	118 (51.5)	0.673	Female	8 (61.5)	5 (38.5)	0.349
Male	131 (50.4)	129 (49.6)		Male	39 (47.6)	43 (52.4)	
**Smoking habits**				**Smoking habits**			
Current	69 (41.3)	98 (58.7)		Current	9 (42.9)	12 (57.1)	
Former	139 (54.5)	116 (45.5)	**0.029**	Former	34 (51.5)	32 (48.5)	0.787
Never	34 (50.7)	33 (49.3)		Never	4 (50.0)	4 (50.0)	
**Histology**				**Histology**			
ADC	157 (52.5)	142 (47.5)		ADC	29 (64.4)	16 (35.5)	
SCC	75 (43.9)	96 (56.1)	0.189	SCC	17 (37.0)	29 (63.0)	**0.019**
Other	10 (52.6)	9 (47.4)		Other	1 (25.0)	3 (75.0)	
**Stage (8th Edition)**				**Stage (8th Edition)**			
I	148 (52.5)	134 (47.5)		I	23 (52.3)	21 (47.7)	
II	48 (45.3)	58 (54.7)	0.245	II	14 (45.2)	17 (54.8)	0.459
III	40 (43.5)	52 (56.5)		III	8 (44.4)	10 (55.6)	
IV	6 (66.7)	3 (33.3)		IV	2 (100.0)	0 (0.0)	
**Adjuvant therapy**				**Adjuvant therapy**			
No	157 (49.5)	160 (50.5)	0.992	No	27 (50.0)	27 (50.0)	0.906
Yes	59 (49.6)	60 (50.4)		Yes	20 (48.8)	21 (51.2)	

ADC: adenocarcinoma; SCC: squamous cell carcinoma.

Table S6. Relationship between TMPRSS4 protein levels and clinicopathological characteristics of the patients from the MDA cohort. The top quartile was used as cut-off value to define high vs. low expression.

MDA Cohort (*n* = 489)			
	**TMP Low**	**TMP High**	***p***
**Age**	**n (%)**	**n (%)**	
< 65	132 (73.3)	48 (26.7)	0.503
≥65	235 (76.1)	74 (23.9)	
**Gender**			
Female	184 (70.8)	76 (29.2)	**0.02**
Male	183 (79.9)	46 (20.1)	
**Smoking habits**			
Current	56 (83.6)	11 (16.4)	
Former	119 (78.0)	56 (22.0)	**0.009**
Never	44 (93.6)	3 (6.4)	
**Histology**			
ADC	243 (81.3)	56 (18.7)	
SCC	111 (64.9)	60 (35.1)	**<0.001**
Other	13 (68.4)	6 (31.6)	
**Stage (8th Edition)**			
I	229 (81.2)	53 (18.8)	
II	72 (67.9)	34 (32.1)	**0.001**
III	58 (63.0)	34 (37.0)	
IV	8 (88.9)	1 (11.1)	
**Adjuvant therapy**			
No	247 (77.9)	77 (22.1)	0.474
Yes	82 (68.9)	37 (31.1)	

ADC: adenocarcinoma; SCC: squamous cell carcinoma.

Table S7. Univariable Cox proportional hazards analysis of TMPRSS4 protein expression and RFS or OS in the MDA cohort. Dichotomization of TMPRSS4 expression was based on quartiles (considering the top 25%, Q4 as high). TMPRSS4 was found to be an independent prognostic factor for both RFS and OS. For the multivariable analysis, only variables whose p value was ≤ 0.1 were considered.

MDA Cohort (n=489)						
	RFS			OS		
	HR	95% CI	*p*	HR	95% CI	*p*
**Age**						
< 65	1		0.456	1		
≥65	0.88	(0.65-1.2)		1.61	(1.21-2.16)	**<0.001**
**Gender**						
Female	1		0.727	1		
Male	0.94	(0.70-1.28)		0.65	(0.50-0.86)	**0.002**
**Smoking habits**						
Never	1			1		
Former	1.48	(0.88-2.50)	0.133	1.76	(1.09-2.86)	**0.021**
Current	1.82	(1.06-3.12)	**0.028**	2	(1.21-3.31)	**0.006**
**Histology**						
ADC	1			1		
SCC	1.17	(0.85-1.62)	0.308	1.77	(1.35-2.32)	**<0.001**
Other	1.13	(0.5-2.59)	0.755	1.72	(0.90-3.3)	0.098
**Stage (8th Edition)**						
I	1			1		
II	1.99	(1.35-2.84)	**<0.001**	2.32	(1.69-3.17)	**<0.001**
III-IV	2.81	(1.96-4.02)	**<0.001**	2.59	(1.88-3.56)	**<0.001**
**Adjuvant therapy**						
No	1			1		
Yes	1.35	(0.96-1.90)	0.082	1.1	(0.80-1.5)	0.55
**TMPRSS4**						
Low	1			1		
High	2.09	(1.53-2.87)	**<0.001**	1.82	(1.38-2.41)	**<0.001**

Table S8. Clinicopathologial characteristics of stage IA/IB NSCLC patients from the MDA cohort that were used to study the prognostic value of TMPRSS4.

MDA Cohort Stage I (*n* = 282)	
**Age**	***n* (%)**
<65	101 (35.8)
≥65	181 (64.2)
**Gender**	
Female	143 (50.7)
Male	181 (49.3)
**Smoking habit**	
Current	91 (32.3)
Former	142 (50.4)
Never	46 (16.3)
**Histology**	
ADC	188 (66.7)
SCC	84 (29.8)
Other	10 (3.5)
**Stage (8th Edition)**	
IA1	100 (35.5)
IA2	14 (4.9)
IA3	73 (25.9)
IB	95 (33.7)
**Adjuvant therapy**	
No	230 (81.6)
Yes	23 (8.2)
NA *	29 (10.2)

* Not available; ADC: adenocarcinoma; SCC: squamous cell carcinoma.
